# Dating ancient splits in phylogenetic trees, with application to the human-Neanderthal split

**DOI:** 10.1186/s12863-023-01185-8

**Published:** 2024-01-02

**Authors:** Keren Levinstein Hallak, Saharon Rosset

**Affiliations:** Department of Statistics and Operations Research, School of Mathematical Sciences, Tel-Aviv University, Tel-Aviv, 6997801 Israel

**Keywords:** Divergence times, Time to most recent common ancestor (TMRCA), Mitochondrial DNA (mtDNA), BEAST2, Transition rates, Poisson parity, Ancient humans, Neanderthal, Denisovan, Chimpanzee

## Abstract

**Background:**

We tackle the problem of estimating species TMRCAs (Time to Most Recent Common Ancestor), given a genome sequence from each species and a large known phylogenetic tree with a known structure (typically from one of the species). The number of transitions at each site from the first sequence to the other is assumed to be Poisson distributed, and only the parity of the number of transitions is observed. The detailed phylogenetic tree contains information about the transition rates in each site. We use this formulation to develop and analyze multiple estimators of the species’ TMRCA. To test our methods, we use mtDNA substitution statistics from the well-established Phylotree as a baseline for data simulation such that the substitution rate per site mimics the real-world observed rates.

**Results:**

We evaluate our methods using simulated data and compare them to the Bayesian optimizing software BEAST2, showing that our proposed estimators are accurate for a wide range of TMRCAs and significantly outperform BEAST2. We then apply the proposed estimators on Neanderthal, Denisovan, and Chimpanzee mtDNA genomes to better estimate their TMRCA with modern humans and find that their TMRCA is substantially later, compared to values cited recently in the literature.

**Conclusions:**

Our methods utilize the transition statistics from the entire known human mtDNA phylogenetic tree (Phylotree), eliminating the requirement to reconstruct a tree encompassing the specific sequences of interest. Moreover, they demonstrate notable improvement in both running speed and accuracy compared to BEAST2, particularly for earlier TMRCAs like the human-Chimpanzee split. Our results date the human – Neanderthal TMRCA to be $\sim 408,000$ years ago, considerably later than values cited in other recent studies.

**Supplementary Information:**

The online version contains supplementary material available at 10.1186/s12863-023-01185-8.

## Background

Dating species divergence has been studied extensively for the last few decades using approaches based on genetics, archaeological findings, and radiocarbon dating [1, 2]. Finding accurate timing is crucial in analyzing morphological and molecular changes in the DNA, in demographic research, and in dating key fossils. One approach for estimating the divergence times is based on the molecular clock hypothesis [3, 4] which states that the rate of evolutionary change of any specified protein is approximately constant over time and different lineages. Subsequently, statistical inference can be applied to a given phylogenetic tree to infer the dating of each node up to calibration.

Our work focuses on this estimation problem and proposes new statistical methods to date the TMRCA in a coalescent tree of two species given a detailed phylogenetic tree for one of the species with the same transition rates per site. Our work does not detect introgression events, and in cases of introgression [5] should be used alongside methods for introgression detection (e.g. [6]). We note that dating the TMRCA in a coalescent tree is different than finding the population tree divergence time. A discussion regarding the differences between the two is available in [7, 8]. Specifically, the discordance of nuclear and mtDNA histories [9] suggests the coalescent tree and population tree of humans may have a different topology.

We formulate the problem of dating the TMRCA by modeling the number of transitions ($A\leftrightarrow G,C\leftrightarrow T$) in each site using a Poisson process with a different rate per site; sites containing transversions are neglected due to their sparsity (indeed, we include sparse transversions in the simulations and show that our methods are robust to their occurrences). The phylogenetic tree is used for estimating the transition rates per site. Hence, when considering two representative sequences, one from each population, our problem reduces to a binary sequence where the parity of the number of transitions of each site is the relevant statistic from which we can infer the time difference between them.

We can roughly divide the approaches to solving this problem into two. The **frequentist** approach seeks to maximize the likelihood of the observed data. Most notable is the PAML [10] package of programs for phylogenetic analyses of DNA and the MEGA software [11]. Alternatively, the **Bayesian** approach considers a prior of all the problem’s parameters and maximizes the posterior distribution of the observations. Leading representatives of the Bayesian approach are BEAST2 [12] and MrBayes [13], which are publicly available programs for Bayesian inference and model choice across a wide range of phylogenetic and evolutionary models.

In this work, we developed several distinct estimators from frequentist and Bayesian approaches to find the TMRCA directly. The proposed estimators differ in their assumptions on the generated data, the approximations they make, and their numerical stability. We explain each estimator in detail and discuss its properties.

A critical difference between our proposed solutions and existing methods is that we seek to estimate only one specific problem parameter. At the same time, software packages such as BEAST2 and PAML optimize over a broad set of unknown parameters averaging the error on all of them (the tree structure, the timing of every node, the per-site substitution rates, etc.). Subsequently, the resources they require for finding a locally optimal instantiation of the tree and dating all its nodes can be very high in terms of memory and computational complexity. Consequently, the number of sequences they can consider simultaneously is highly limited. Thus, unlike previous solutions, we utilize transition statistics from all available sequences, in the form of a previously built phylogenetic tree.

We develop a novel approach to simulate realistic data to test our proposed solutions. To do so, we employ Phylotree [14, 15] – a complete, highly detailed, constantly updated reconstruction of the human mitochondrial DNA phylogenetic tree. In our work we assume all substitutions are specified by Phylotree (for an elaborate discussion regarding the correction of this claim see [16]). We sample transitions of similar statistics to Phylotree and use it to simulate a sequence at a predefined trajectory from Phylotree’s root.

We then empirically test the different estimators on simulated data and compare our results to the BEAST2 software. Our proposed estimators are calculated substantially faster while utilizing the transitions statistics from all available sequences (Phylotree considers 24,275 sequences), unlike BEAST2 which can consider only dozens of sequences due to its complexity. Comparing with the ground truth, we show that BEAST2 slightly overestimates the TMRCA, while our estimates provide more accurate results. For larger TMRCAs such as the human-Chimpanzee, BEAST2 also has a larger variance compared to our methods. Finally, we use our estimators to date the TMRCA (given in kya – kilo-years ago) of modern humans with Neanderthals, Denisovan and Chimpanzee based on their mtDNA. Surprisingly, the TMRCAs we find (human-Neanderthals $\sim$408 kya, human-Denisovans $\sim$841 kya, human-Chimpanzee $\sim$5,010 kya) – are considerably later than those accepted today.

## Methods

### Estimation methods

First, we describe an idealized reduced mathematical formulation for estimating TMRCAs and our proposed solutions. In Estimating ancient TMRCAs using a large modern phylogeny section, we describe the reduction process in greater detail.

Consider the following scenario: we have a set of *n* Poisson rates, denoted as $\{{\lambda _i}\}_{i=1}^n$ where $n \in \mathbb {N}$. Let $\vec {X}$ be a vector of length *n* such that each element $X_i$ is independently distributed as $\text {Pois}(\lambda _i)$. Similarly, let $\vec {Y}$ be a vector of length *n* such that each element $Y_i$ is independently distributed as $\text {Pois}(\lambda _i \cdot p)$ for a fixed unknown *p*. We denote $\vec {Z}$ as the coordinate-wise parity of $\vec {Y}$, meaning that $Z_i = 1$ if $Y_i$ is odd and $Z_i = 0$ otherwise. **Our goal is to estimate** *p* **given** $\vec {X}$ **and** $\vec {Z}$.

#### Remark 1

Note that the number of *unknown* Poisson rate parameters *n* in the problem $\{\lambda _i\}_{i=1}^n$ grows with the number of observations $\{(X_i, Z_i)\}_{i=1}^n$. However, our focus is solely on estimating *p*, so additional observations do provide more information.

#### Remark 2

The larger the value of $p \cdot \lambda _i$, the less information on *p* is provided in $Z_i$ as it approaches a Bernoulli distribution with a probability of 0.5. On the other hand, the smaller $\lambda _i$ is, the harder it will be to infer $\lambda _i$ from $X_i$. As a result, the problem of estimating *p* should be easier in settings where $\lambda _i$ is high and *p* is low.

#### Preliminaries

First, we derive the distribution of $Z_i$; All proofs are provided in the Supplementary material (Section 1).

##### Lemma 1

Let $Y \sim \text {Pois}(\Lambda )$ and *Z* be the parity of *Y*. Then $Z \sim Ber(\frac{1}{2}(1-e^{-2\Lambda }))$.

We use this result to calculate the likelihood and log-likelihood of *p* and $\vec \lambda$ given $\vec {X}$ and $\vec {Z}$. The likelihood is given by:

1 $$\begin{aligned} L\left( {\vec X,\vec Z;p,\vec \lambda } \right) = \prod \limits _{i = 1}^n {{e^{ - {\lambda _i}}}\frac{{{\lambda _i}^{{X_i}}}}{{{X_i}!}}\frac{1}{2}\left( {1 + {{\left( { - 1} \right) }^{{Z_i}}}{e^{ - 2{\lambda _i}p}}} \right) }, \end{aligned}$$

and the log-likelihood is:

2 $$\begin{aligned} l\left( {\vec X,\vec Z;p,\vec \lambda } \right) = \sum \limits _{i = 1}^n {\left[ { - {\lambda _i} + {X_i}\log {\lambda _i} + \log \left( {1 + {{\left( { - 1} \right) }^{{Z_i}}}{e^{ - 2{\lambda _i}p}}} \right) } \right] + Const.} \end{aligned}$$

This result follows immediately from the independence of each coordinate.

#### Cramer-Rao bound

We begin our analysis by computing the Cramer-Rao bound (CRB; [17, 18]). In Comparative study on raw simulations section, we compare the CRB to the error of the estimators.

##### Theorem 1

Denote the Fisher information matrix for the estimation problem above by $I \in \mathbb {R}^{(n+1, n+1)}$, where the first *n* indexes correspond to $\{\lambda _i\}_{i=1}^n$ and the last index $(n+1)$ corresponds to *p*. For clarity denote $I_{p, p} \doteq I_{n+1,n+1}, I_{i, p} \doteq I_{i,n+1}, I_{p, i} \doteq I_{n+1,i}$. Then:

3 $$\begin{aligned} \forall i\ne j,\; 1\le i,j\le n: \quad&{I_{i,j}} = 0 ,\; \quad {I_{i,i}} = \frac{1}{{{\lambda _i}}} + \frac{4{p^2}}{e^{ 4{\lambda _i}p} - 1}, \quad {I_{i,p}} = {I_{p,i}} = \frac{4p{\lambda _i}}{e^{ 4{\lambda _i}p} - 1}, \nonumber \\&{I_{p,p}} = 4\sum \limits _{i = 1}^n {\frac{\lambda _i^2}{e^{ 4{\lambda _i}p} - 1}}. \end{aligned}$$

Consequently, an unbiased estimator $\hat{p}$ holds:

4 $$\begin{aligned} \mathbb {E}\left[ (p-\hat{p})^2\right] \ge \left[ 4\sum \limits _{i = 1}^n {\frac{ \lambda _i^2}{ e^{4\lambda _i p} - 1 + 4p^2 \lambda _i }} \right] ^{-1}. \end{aligned}$$

If $\forall i=1..n: \lambda _i=\lambda$, we can further simplify the expression:

5 $$\begin{aligned} \mathbb {E}\left[ (p-\hat{p})^2\right] \ge \frac{e^{4\lambda p} - 1 + 4p^2 \lambda }{4n\lambda ^2 }. \end{aligned}$$

The CRB, despite its known looseness in many problems, provides insights into the sensitivity of the error to each parameter. This expression supports our previous observation that the error of an unbiased estimator increases exponentially with $\min _i \{ \lambda _i \cdot p \}$. However, for constant $\lambda _i \cdot p$, the error improves for higher values of $\lambda _i$. We now proceed to describe and analyze several estimators for *p*.

#### Method 1 - maximum likelihood estimator

##### Proposition 1

Following equation 1, the maximum likelihood estimators $\hat{p}, \hat{\lambda }_i$ hold:

6 $$\begin{aligned} \sum \limits _{i = 1}^n {{{\hat{\lambda }}_i} = } \sum \limits _{i = 1}^n {{X_i}} , \quad {X_i} = {{\hat{\lambda }}_i} + \frac{2\hat{p}{{\hat{\lambda }}_i}}{ {{{\left( { - 1} \right) }^{{Z_i}}}{e^{ 2{{\hat{\lambda }}_i}\hat{p}}}} + 1}, \quad \sum \limits _{i=1}^n {\frac{\hat{\lambda }_i}{{ { {{\left( { - 1} \right) }^{{Z_i}}}{e^{ 2{{\hat{\lambda }}_i}\hat{p}}} + 1} }}} = 0. \end{aligned}$$

Proposition 1 provides *n* separable equations for maximum likelihood estimation (MLE). Our first estimator sweeps over values of $\hat{p}$ (grid searching in a relevant area) and then for each $i=1..n$ finds the optimal $\hat{\lambda }_i$ numerically. The solution is then selected by choosing the pair $(\hat{p}, \{\hat{\lambda }_i\}_{i=1}^n)$ that maximizes the log-likelihood calculated using equation 2.

The obtained MLE equations are solvable, yet, finding the MLE still requires solving *n* numerical equations, which might be time-consuming. More importantly, MLE estimation is statistically problematic when the number of parameters is of the same order as the number of observations [19]. Subsequently, we propose alternative methods that may yield better practical results.

#### Method 2 - $\lambda _i$-conditional estimation

We propose a simple estimate of $\vec {\lambda }$ based solely on $X_i$, followed by an estimate of *p* as if $\vec {\lambda }$ is known, considering only $\vec {Z}$. This method is expected to perform well when $\lambda _i$ values are large, as in these cases, $X_i$ conveys more information about $\lambda _i$ than $Z_i$. This approach enables us to avoid estimating both $\vec {\lambda }$ and *p* simultaneously, leading to a simpler numerical solution.

When $p \le 1$, we can mimic $Y_i$’s distribution as a sub-sample from $X_i$, i.e. we assume that $Y_i|X_i \sim Bin(n=X_i,p)$. Then, we find the maximum likelihood estimate of *p*:

##### Proposition 2

If $$Y_i|X_i \sim Bin(X_i,p)$$, then:

1. $Y_i \sim \text {Pois} (\lambda _i \cdot p)$, which justifies this approach.
2. $Z_i|X_i \sim Ber\left( {\frac{1}{2}\left( {1 - {{\left( {1 - 2p} \right) }^{{X_i}}}} \right) } \right)$, so we can compute the likelihood of *p* without considering $\lambda _i$.
3. The maximum likelihood estimate of *p* given $\sum \limits _{i=1}^n Z_i$ holds:
  7 $$\begin{aligned} \sum \limits _{i = 1}^n {{\left( {1 - 2\hat{p}} \right) }^{X_i}} = n - 2\sum \limits _{i=1}^n {Z_i} \end{aligned}$$

##### Remark 3

We use the maximum likelihood estimation of *p* given $\sum _{i=1}^n Z_i$ by applying Le-Cam’s theorem [20]. This eliminates the need for a heuristic solution of the pathological case $X_i=0, Z_i=1$.

#### Method 3 - Gamma distributed Poisson rates

The Bayesian statistics approach incorporates prior assumptions about the parameters. A common prior for the rate parameters $\vec {\lambda }$ is the Gamma distribution, which is used in popular Bayesian divergence time estimation programs such as MCMCtree [10], BEAST2 [12], and MrBayes [13]. Specifically, we have $\lambda _i \sim \Gamma (\alpha ,\beta )$, and for *p*, we use a uniform prior over the positive real line.

##### Proposition 3

Let $\lambda _i \sim \Gamma (\alpha ,\beta )$, then the maximum a posteriori estimator of *p* holds:

8 $$\begin{aligned} \frac{{\partial l}}{{\partial p}} = \sum \limits _{i=1}^n{\frac{X_i + \alpha }{{{\left( { - 1} \right) }^{{Z_i}}}{{\left( 1 + {\frac{2p}{{\beta + 1}}} \right) }^{X_i + \alpha }}+1}}=0 \end{aligned}$$

Subsequently, given estimated values for $\alpha$ and $\beta$, we can find an estimator for *p* numerically to hold Equation 8. Unfortunately, the derivative with respect to $\alpha$ does not have a closed-form expression, nor is it possible to waive the dependence on $\vec {Z}, p$. Hence, we suggest using Negative-Binomial regression [21] to estimate $\alpha$ and $\beta$ given $\vec {X}$ .

### Estimating ancient TMRCAs using a large modern phylogeny

In this section, we apply the methods described in Estimation methods section to estimate the non-calibrated TMRCAs of humans and their closest relatives by comparing mitochondrial DNA (mtDNA) sequences. Our approach assumes the following assumptions:

1. Molecular clock assumption - the rate of accumulation of transitions (base changes) over time and across different lineages is constant, as first proposed by Zuckerkandl and Pauling [3] and widely used since.
2. Poisson distribution - The number of transitions along the human and human’s closest relatives mtDNA lineages follows a Poisson distribution with site-dependent rate parameter $\lambda _i$ per time unit (implying that the rate of accumulation of transitions is independent of the time since the last transition).
3. No transversions - We only consider sites with no transversions and assume a constant transition rate per site ($\lambda _{i,A \rightarrow G}=\lambda _{i,G \rightarrow A}$, or $\lambda _{i,T \rightarrow C}=\lambda _{i,C \rightarrow T}$).
4. Independence of sites - The number of transitions at each site is independent of those at other sites.
5. Phylogenetic tree - The phylogenetic tree presented in the Phylotree database includes all transitions and transversions that occurred along the described lineages.

As the Phylotree database is based on tens of thousands of sequences, the branches in the tree correspond to relatively short time intervals, making multiple mutations per site unlikely in each branch [22]. However, when considering the mtDNA sequence of other species, the branches in the tree correspond to much longer time intervals, meaning that many underlying transitions are unobserved. For instance, when comparing two human sequences that differ in a specific site, Phylotree can determine whether the trajectory between the sequences was $A \rightarrow G$, $A \rightarrow G \rightarrow A \rightarrow G$, or $A \rightarrow T \rightarrow G$. However, when comparing sequences of ancient species, an elaborate phylogenetic tree like Phylotree is not available, making it impossible to discriminate between these different trajectories.

We use the following notation:

1. Let $\vec {X}_{\text {mtDNA}}$ denote the number of transitions observed at each site along the human mtDNA phylogenetic tree as described by Phylotree. Each coordinate corresponds to a different site out of the 16,569 sites. The number of transitions at site *i*, $X_{\text {mtDNA,i}}$, follows a Poisson distribution with parameter $\lambda _i$.
2. Let $\vec {Y}$ denote the number of transitions between two examined sequences (e.g. a modern human and a Neanderthal). We normalize the length of the tree edges so that the sum of all Phylotree’s edges is one. The estimated parameter *p* relates to the edge distance between the two examined sequences. Subsequently, $Y_i$ follows a Poisson distribution with parameter $\lambda _i \cdot p$.
3. Let $\vec {Z}$ denote the parity of $\vec {Y}$.

**Fig. 1:**
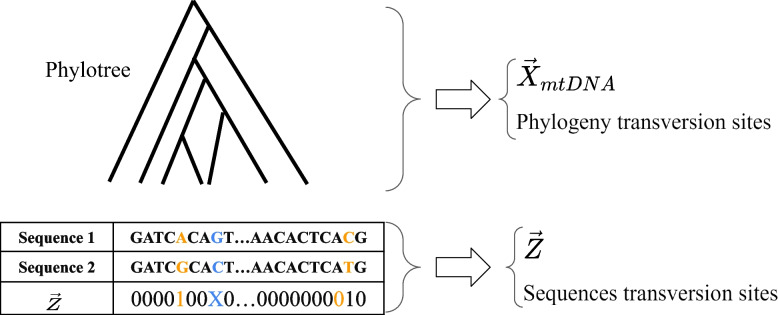
We use a large, comprehensive phylogeny, such as Phylotree, and assume its tree topology and branch lengths are known. We also assume that the phylogeny is detailed enough so that it describes all the substitutions that occurred between its sequences. From this detailed phylogeny, we extract a list of the number of transitions and transversions that occurred in each site along the phylogeny to get $\vec {X}_{mtDNA}$ (composed of the number of transitions that occurred at each site) and a list of phylogeny transversion sites - sites in which at least one transversion occurred along the phylogeny. We aim to estimate the distance between two sequences that are not necessarily part of the tree and may be much more distant than branch tree lengths. To do so, we extract a binary vector $\vec {Z}$ that states for each site whether the sequences are identical ($z_i=0$, marked black) or different ($z_i=1$, marked orange). We check in which sites a transversion must have occurred between the two sequences (marked blue) and remove these sites from $\vec {X}_{mtDNA}$ and $\vec {Z}$, thus shortening these vectors. We do the same for the phylogeny transversion sites

Using $\vec {X}$ and $\vec {Z}$, we can estimate *p* using the methods in Estimation methods section. The TMRCA is given by: $\frac{1}{2}(T_{\text {sequence 1}} + T_{\text {sequence 2}} + p)$ when $T_{\text {sequence 1,2}}$ are the estimated times of the examined sequences measured in (uncalibrated) units of phylotree’s total tree length. For clarity, we summarize the process described in this section in Fig. 1.

### Calibration

Our methods output *p*, which is the ratio of two values:

1. The sum of the edges between the two examined sequences and their most recent common ancestor (MRCA).
2. The total sum of Phylotree’s edges.

Similarly to BEAST2, to calibrate *p* to years, we use the per-site per-year substitution rate for the coding region given in [23] $\mu =$ 1.57 x 10E-8. We then calculate the total sum of Phylotree’s edges in years by dividing the average number of substitutions in the coding region per site (1.4) by $\mu$.

## Results

### Comparative study on raw simulations

**Fig. 2:**
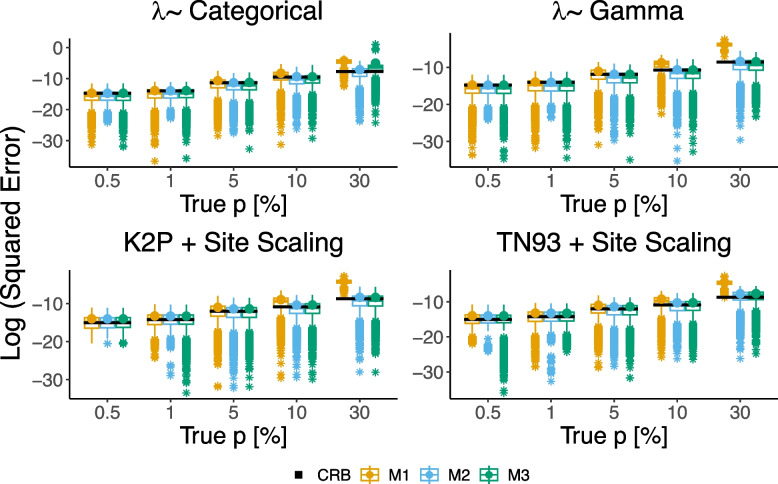
Box-plot of the log squared estimation errors of the three proposed methods for selected values of *p*, expressed as a percentage of the total length of Phylotree’s edges (outliers are marked with $*$). The simulations were run 10, 000 times for each value of *p*. The CRB is shown in black for reference and the circles represent the log of the mean values which are comparable to the CRB. The experiments were conducted for two different distributions of $\lambda$ and two different substitution models: (Top left) Categorical distribution with two values: $\epsilon =0.1$ with probability $\eta =0.11$ and $a=11.87$ with probability $1-\eta$.(Top right) Gamma distribution with parameters $\alpha$ and $\beta$. (Bottom left) Site-scaled K2P substitution model. (Bottom right) Site-scaled TN93 substitution model

To compare the performance of the three estimation methods described in Estimation methods section, we conducted experiments using simulated data. The Poisson rates $\lambda$ were generated to reflect the substitution rates observed in mtDNA data using either a Categorical or a Gamma distribution. The parameters for the Gamma distribution ($\alpha =0.23, \beta =0.164$) were estimated directly from the data, while the parameters for the Categorical distribution were chosen such that both distributions have the same mean and variance. One of the Categorical values ($\epsilon =0.1$) corresponds to the rate of low activity sites in the mtDNA data. The other value ($a=11.87$) and the probabilities (0.11, 0.89) were chosen accordingly. To test the robustness of our methods, we have also conducted simulations using the celebrated K2P [24] and TN93 [25] substitution models, with rate matrix parameters and site scaling extracted from Phylotree’s data. Further simulation details appear in the Supplementary material, Section 2.1. The comparison results are shown in Fig. 2 with the Cramer-Rao bound for reference. To provide a qualitative comparison, we performed a one-sided paired Wilcoxon signed rank test on every pair of models, correcting for multiple comparisons using the Bonferroni correction. Our results show that Method 2 has the lowest squared error while Method 1 has the highest squared error, for all distributions and substitution models. It is noteworthy that although Method 3 assumes a Gamma distribution, it still performs well even when a model mismatch exists.

### Phylogenetic tree simulations

We validated our methods by testing their performance in a more realistic scenario of simulating a phylogenetic tree. Our methods take as input the observed transitions along Phylotree ($\vec {X}_{\text {mtDNA}}$) using all of its 24,275 sequences and a binary vector $\vec {Z}$ denoting the differences between two sequences, which we aim to estimate the distance between. We compared our methods to the well-known BEAST2 software [12], which, similarly to other well-established methods (such as MCMCtree [10], MrBayes [13], etc.) considers sequences along with their phylogenetic tree to produce time estimations. The software BEAST2 performs Bayesian analysis using MCMC to average over the space of possible trees. However, it is limited in its computational capacity, so it cannot handle a large number of sequences like those in Phylotree. For this reason, we used a limited set of diverse sequences, including mtDNA genomes of 53 humans [26], the revised Cambridge Reference Sequence (rCRS) [27], the root of the human phylogenetic mtDNA tree, termed Reconstructed Sapiens Reference Sequence (RSRS) [28], and 10 ancient modern humans [23]. More details about the parameters used by BEAST2 are available in the Supplementary material, Section 2.3. To evaluate our methods, we added a simulated sequence with a predefined distance from the RSRS.

Our aim is to generate a vector $\vec {\lambda }$ that produces a vector $\vec {X}$ that has a similar distribution to $\vec {X}{\text {mtDNA}}$. The human mtDNA tree has 16,569 sites, of which 15,629 have no transversions. The MLE of $\lambda _i$ at each site is the observed number of transitions, $X_{\text {mtDNA,i}}$. However, simulating $\vec {\lambda }$ as $\vec {X}_{\text {mtDNA}}$ leads to an undercount of transitions because 10,411 sites ($67\%$ of the total number of sites considered) had no transitions along the tree and their Poisson rate is taken to be zero. To mitigate this issue, the rates for these sites were chosen to be $\epsilon$, the value that minimizes the Kolmogorov-Smirnov statistic [29, 30] (details are provided in the Supplementary material, Section 2.2).

**Fig. 3:**
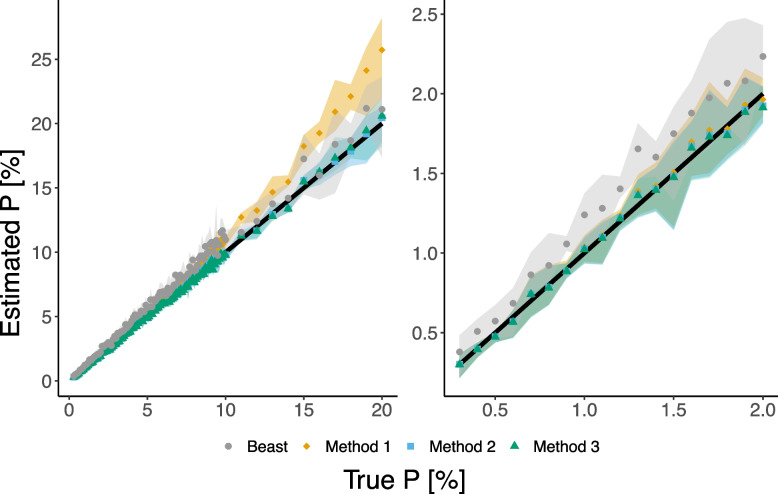
Comparison of our methods with BEAST2 estimator using simulated data. The right plot shows a zoom-in view of the left plot, focusing on values of *p* between 0 and $20\%$. Each point in the plot represents the average of 5 runs, while the shaded regions indicate the range of estimations obtained. We note that in order to compare to our methods we only present here point estimates for BEAST2 (posterior means) and not the HPD of the full posterior distribution

The results are presented in Fig. 3. Similarly to Fig. 2, Method 1 has a larger error than Methods 2 and 3 for values of *p* within the simulated region, and the gap widens with increasing *p*. Methods 2 and 3 provide the best results for the entire range of p. Compared to Methods 2 and 3, BEAST2 is less accurate and has a larger variance for higher values of p. Additionally, BEAST2 has a much longer running time (roughly 1 hour) compared to our methods (less than a second). BEAST2 simulation presented here was conducted using a fixed tree topology. The results for a simulation without a fixed tree topology (running time $\sim 1.5$ hours) are presented in Supplementary Fig. 2. Furthermore, to test the effect of additional sequences, we conducted simulations with an additional 50 human sequences for selected values of *p* and a fixed tree topology (running time $\sim 3$ hours). Supplementary Fig. 3 presents a comparison of estimation errors for various BEAST2 estimators compared to Method 2. Finally, to examine the effect of removing transversion sites, we conducted simulations with different ti/tv ratios showing that decreasing the ti/tv ratio does not result in bias (Supplementary Fig. 4).

### Real data results

**Fig. 4:**
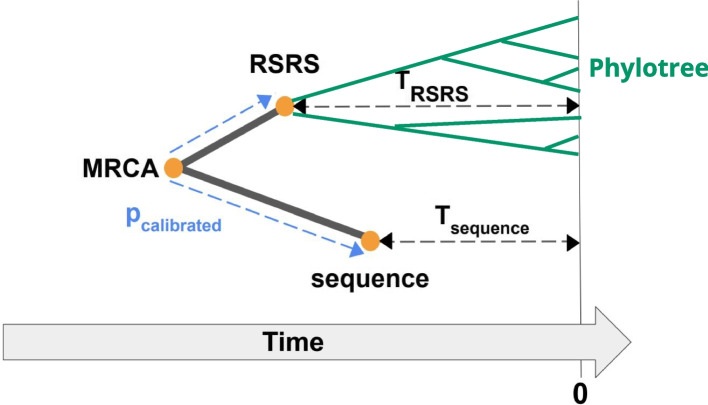
We used the following schema to obtain estimates for the time distance between real-world sequences: We first extracted $\vec {X}_{mtDNA}$ from Phylotree. Then, we determined the binary vector $\vec {Z}$ that denotes the differences between the RSRS and the sequence in consideration. We removed from both $\vec {X}_{mtDNA}$ and $\vec {Z}$ the phylogeny transversion sites and sequences transversion sites as explained in Fig. 1. We applied our estimation methods using $\vec {X}_{mtDNA}$ and $\vec {Z}$ as input to get uncalibrated *p* and calibrated *p* as explained in Calibration section. Finally, the TMRCA of the examined sequence and humans is given by $TMRCA = \frac{1}{2}(T_{RSRS}+T_{Sequence}+p_{calibrated})$

As the final step of our experiments, we apply our methods to real-world data to determine the TMRCA of the modern human and Neanderthal, Denisovan, and chimpanzee mtDNA genomes. A schema of our estimation process is provided in Fig. 4. Table 1 displays the uncalibrated distances between modern humans and each sequence, compared to the estimates from BEAST2. The presented TMRCA represents an average of the TMRCA obtained from 55 modern human mtDNA sequences of diverse origins [26]. Table 2 presents the TMRCA in kya (kilo-years ago) of the modern human and each sequence. We note that if some of the modern human sequences were very close to one another, a weighted average would be more appropriate, considering the proximity of sequences and giving sequences closer to one another a smaller weight. As the sequences in our case are from diverse origins, a uniform average is a good approximation. An alternative strategy to calculating the average TMRCA with all human mtDNA sequences is to use the RSRS instead. Moreover, we can use the MRCA of Neanderthals instead of using all Neanderthal sequences and the same for any other population with a confidently known MRCA, resulting in one estimate instead of multiple pairwise estimates. This strategy involves combining different possibly noisy TMRCAs (one for each population and one for the distance between the populations).

**Table 1:** Uncalibrated distances between modern humans and selected hominins

Sample	BEAST2	Method 1	Method 2	Method 3
**Altai**	0.98 (±0.08)	0.8 (±0.08)	0.79 (±0.08)	0.79 (±0.08)
**Denisova15**	0.99 (±0.08)	0.81 (±0.08)	0.8 (±0.08)	0.8 (±0.08)
**HST**	0.99 (±0.07)	0.78 (±0.08)	0.78 (±0.08)	0.77 (±0.08)
**Mezmaiskaya1**	1.03 (±0.08)	0.86 (±0.09)	0.85 (±0.09)	0.85 (±0.09)
**Chagyrskaya08**	1.04 (±0.08)	0.84 (±0.09)	0.83 (±0.09)	0.83 (±0.08)
**ElSidron1253**	1.06 (±0.08)	0.83 (±0.08)	0.82 (±0.08)	0.82 (±0.08)
**Vindija33.17**	1.08 (±0.08)	0.86 (±0.09)	0.85 (±0.09)	0.85 (±0.09)
**Feldhofer1**	1.09 (±0.08)	0.85 (±0.09)	0.84 (±0.08)	0.83 (±0.08)
**GoyetQ56-1**	1.09 (±0.08)	0.88 (±0.09)	0.88 (±0.09)	0.87 (±0.09)
**GoyetQ57-2**	1.09 (±0.08)	0.84 (±0.09)	0.83 (±0.08)	0.83 (±0.08)
**Les Cottes Z4-1514**	1.09 (±0.08)	0.91 (±0.09)	0.91 (±0.09)	0.9 (±0.09)
**Mezmaiskaya2**	1.09 (±0.08)	0.84 (±0.09)	0.83 (±0.09)	0.83 (±0.08)
**Vindija33.16**	1.09 (±0.08)	0.87 (±0.09)	0.86 (±0.09)	0.86 (±0.09)
**Vindija33.25**	1.09 (±0.08)	0.85 (±0.09)	0.84 (±0.09)	0.83 (±0.09)
**GoyetQ305-7**	1.09 (±0.08)	0.89 (±0.09)	0.89 (±0.09)	0.88 (±0.09)
**GoyetQ374a-1**	1.09 (±0.08)	0.89 (±0.09)	0.89 (±0.09)	0.88 (±0.09)
**Spy 94a**	1.09 (±0.08)	0.88 (±0.09)	0.88 (±0.09)	0.87 (±0.09)
**Sima de los Huesos**	1.79 (±0.11)	1.42 (±0.12)	1.39 (±0.11)	1.39 (±0.11)
**Denisova2**	2.02 (±0.12)	1.68 (±0.13)	1.65 (±0.12)	1.64 (±0.12)
**Denisova8**	2.06 (±0.12)	1.69 (±0.13)	1.66 (±0.13)	1.65 (±0.12)
**Denisova4**	2.18 (±0.12)	1.83 (±0.14)	1.79 (±0.13)	1.78 (±0.13)
**Denisova3**	2.19 (±0.12)	1.82 (±0.13)	1.78 (±0.13)	1.77 (±0.13)
**Chimpanzee**	16.33 (±1.22)	12.75 (±0.68)	11.21 (±0.53)	11.21 (±0.53)

Uncalibrated distances expressed as a percentage of the total length of Phylotree’s edges, as determined by our methods compared with BEAST2. The values correspond to *p*, and indicate the estimation’s location in Fig. 3. In the parentheses, we provide the standard deviation for each estimator, obtained from bootstrapping 100 site samples for every modern human – ancient sequence pair in the dataset. Note that the BEAST2 values presented here were de-calibrated as described in Calibration section

**Table 2:** Estimated TMRCAs of modern human and selected hominins

Sample	BEAST2	Method 1	Method 2	Method 3
**Altai**		425.75 (±39.9)	423.51 (±39.47)	421.09 (±39.02)
**Denisova15**		427.61 (±39.38)	425.13 (±38.91)	422.75 (±38.54)
**HST**		414.19 (±40.78)	411.68 (±40.34)	409.72 (±39.95)
**Mezmaiskaya1**		430.56 (±41.13)	428.13 (±40.6)	425.64 (±40.28)
**Chagyrskaya08**		416.76 (±39.93)	414.23 (±39.36)	411.74 (±39.07)
**ElSidron1253**		403.37 (±38.1)	400.94 (±37.58)	398.43 (±37.23)
**Vindija33.17**		410.96 (±39.74)	408.44 (±39.19)	406.05 (±38.86)
**Feldhofer1**		399.36 (±38.45)	396.93 (±37.91)	394.37 (±37.59)
**GoyetQ56-1**		416.06 (±39.76)	413.6 (±39.11)	411.1 (±38.86)
**GoyetQ57-2**		394.71 (±38.28)	392.28 (±37.78)	389.72 (±37.43)
**Les Cottes Z4-1514**		428.91 (±41.04)	426.3 (±40.42)	423.95 (±40.07)
**Mezmaiskaya2**		395.99 (±38.64)	393.58 (±38.11)	391.02 (±37.74)
**Vindija33.16**		409.84 (±39.4)	407.47 (±38.81)	404.79 (±38.53)
**Vindija33.25**		399.91 (±39.27)	397.48 (±38.74)	394.92 (±38.4)
**GoyetQ305-7**		418.16 (±40.35)	415.45 (±39.69)	413.3 (±39.4)
**GoyetQ374a-1**		418.16 (±39.72)	415.45 (±39.07)	413.3 (±38.8)
**Spy 94a**		415.03 (±40.47)	412.57 (±39.83)	410.07 (±39.55)
**Humans-Neandertals**	**507.13 (** $\varvec{\pm }$ **33.86)**	**410.46 (** $\varvec{\pm }$ **37.12)**	**408.02 (** $\varvec{\pm }$ **36.78)**	**405.67 (** $\varvec{\pm }$ **36.29)**
**Sima de los Huesos**		850.67 (±66.75)	840.25 (±65.49)	837.38 (±65.06)
**Denisova2**		866.15 (±60.08)	851.42 (±57.98)	847.86 (±57.63)
**Denisova8**		850.67 (±61.93)	835.27 (±59.65)	832.22 (±59.4)
**Denisova4**		860.43 (±62.55)	843.23 (±60.36)	838.71 (±59.91)
**Denisova3**		853.07 (±60.73)	836.1 (±58.52)	831.41 (±58.13)
**Humans-Denisovans-Sima**	**1,017.98 (** $\varvec{\pm }$ **53.56)**	**856.2 (** $\varvec{\pm }$ **54.75)**	**841.26 (** $\varvec{\pm }$ **52.89)**	**837.52 (** $\varvec{\pm }$ **52.29)**
**Humans-Chimpanzee**	**7,292.72 (** $\varvec{\pm }$ **545.48)**	**5,693.51 (** $\varvec{\pm }$ **302.59)**	**5,009.78 (** $\varvec{\pm }$ **235.05)**	**5,005.39 (** $\varvec{\pm }$ **237.13)**

The table displays the estimated TMRCAs (in kya) between modern humans and selected hominins, as determined by our methods and compared with BEAST2. The standard deviation, which arises from a combination of the standard deviation of our methods and the sample dating, is given in parentheses. It’s important to note that BEAST2 calculates the TMRCA for all sequences in the same clade as a single estimate, while our methods estimate the TMRCA for each sample individually by taking the average of estimations derived from comparing the sample with every modern human sequence in the dataset

The estimates from real-world sequences presented in Table 1 are consistent with those obtained for the simulated dataset in Phylogenetic tree simulations section. For low values of *p*, our three methods all produce similar estimates while BEAST2’s has a slightly higher estimate. For the human-Chimpanzee uncalibrated distance, which is relatively high, Method 1 provides a higher estimate than that obtained by Methods 2 and 3, and BEAST2 provides a substantially higher estimate. The results in Table 2 show the TMRCA estimates, which are significantly smaller for our methods than those obtained from BEAST2 for human-Neanderthals and human-Denisovans. For example, BEAST2 estimated the human – Sima de los Huesos – Denisovans TMRCA as $\sim 1,018$ kya, while our best-performing method (2) estimated it as $\sim 841$ kya. This TMRCA is estimated as (540-1,410 kya) in [31]. Similarly, BEAST2 estimated the human – Neanderthal TMRCA as $\sim 507$ kya, while our methods estimated it as $\sim 408$ kya. Preceding literature estimates this time closer to ours ($\sim$400 kya [32–34]) while recent literature provides a much earlier estimate ($\sim$800 kya [35]). Finally, BEAST2 estimates the human-Chimpanzee TMRCA as $\sim 7,293$ kya whereas our estimate is $\sim$5,010 kya, both are close to the literature value of 5 – 8 million years ago [36–39].

## Conclusions

We investigated an estimation problem arising in statistical genetics when estimating the TMRCA of species. The problem’s formulation, estimating Poisson rates from parity samples, leads to multiple estimators with varying assumptions. We calculated the CRB for this estimation problem and compared our methods against commonly used BEAST2 in different empirical settings, including a simple sampling scheme (Comparative study on raw simulations section), a more elaborate generative scheme based on real-world mtDNA data (Phylogenetic tree simulations section), and the calculation of the TMRCA of modern humans and other hominins using their mtDNA genomes (Real data results section).

Our results indicate that our proposed methods are significantly faster and more accurate than BEAST2, especially for earlier TMRCAs such as the human-Chimpanzee. Our methods utilize the transition statistics from the entire known human mtDNA phylogenetic tree (Phylotree) without the need for reconstructing a tree containing the sequences of interest. Our results show that the human – Neanderthal TMRCA is $\sim 408,000$ years ago, considerably later than the values obtained by BEAST2 ($\sim 507,000$ years ago) and other values cited in the literature.

### Supplementary Information

**Additional file 1.**

## Data Availability

The code used in this work is available at: https://github.com/Kerenlh/DivergenceTimes.

## References

[1] Dos Reis M, Donoghue PC, Yang Z (2016). Bayesian molecular clock dating of species divergences in the genomics era. Nat Rev Genet..

[2] Taylor, RE. Radiocarbon dating in archaeology. Encyclopedia of Global Archaeology. Cham: Springer International Publishing; 2020. p. 9050-9060.

[3] Zuckerkandl E, Pauling L, Kasha M, Pullman B. Horizons in biochemistry. Horizons in biochemistry. 1962;97–166.

[4] Zuckerkandl E, Pauling L. In Evolving Genes and Proteins, ed. by V. Bryson & HJ Vogel. New York: Academic Press; 1965. 10.1126/science.147.3653.68 17799782

[5] Posth C, Wißing C, Kitagawa K, Pagani L, van Holstein L, Racimo F (2017). Deeply divergent archaic mitochondrial genome provides lower time boundary for African gene flow into Neanderthals. Nat Commun..

[6] Pickrell J, Pritchard J. Inference of population splits and mixtures from genome-wide allele frequency data. Nat Precedings. 2012;1-1. 10.1371/journal.pgen.1002967 PMC3499260 23166502

[7] Pettengill JB (2015). The time to most recent common ancestor does not (usually) approximate the date of divergence. PLoS ONE..

[8] Sağlam İK, Baumsteiger J, Miller MR (1860). Failure to differentiate between divergence of species and their genes can result in over-estimation of mutation rates in recently diverged species. Proc R Soc B Biol Sci..

[9] Reich D, Green RE, Kircher M, Krause J, Patterson N, Durand EY (2010). Genetic history of an archaic hominin group from Denisova Cave in Siberia. Nature..

[10] Yang Z (2007). PAML 4: phylogenetic analysis by maximum likelihood. Mol Biol Evol..

[11] Kumar S, Tamura K, Nei M (1994). MEGA: molecular evolutionary genetics analysis software for microcomputers. Bioinformatics..

[12] Bouckaert R, Heled J, Kühnert D, Vaughan T, Wu CH, Xie D (2014). BEAST 2: a software platform for Bayesian evolutionary analysis. PLoS Comput Biol..

[13] Ronquist F, Teslenko M, Van Der Mark P, Ayres DL, Darling A, Höhna S (2012). MrBayes 3.2: efficient Bayesian phylogenetic inference and model choice across a large model space. Syst Biol..

[14] Van Oven M, Kayser M (2009). Updated comprehensive phylogenetic tree of global human mitochondrial DNA variation. Hum Mutat..

[15] Van Oven M (2015). PhyloTree Build 17: Growing the human mitochondrial DNA tree. Forensic Sci Int Genet Suppl Ser..

[16] Levinstein-Hallak K, Tzur S, Rosset S (2018). Big data analysis of human mitochondrial DNA substitution models: a regression approach. BMC Genomics..

[17] Cramer H (1946). Mathematical methods of statistics.

[18] Rao CR (1945). Information and the accuracy attainable in the estimation of statistical parameters. Reson J Sci Educ..

[19] Cox DR, Reid N (1987). Parameter orthogonality and approximate conditional inference. J R Stat Soc Ser B (Methodol)..

[20] Le Cam L (1960). An approximation theorem for the Poisson binomial distribution. Pac J Math..

[21] Hilbe JM. Negative binomial regression. Cambridge: Cambridge University Press; 2011.

[22] Soares P, Ermini L, Thomson N, Mormina M, Rito T, Röhl A (2009). Correcting for purifying selection: an improved human mitochondrial molecular clock. Am J Hum Genet..

[23] Fu Q, Mittnik A, Johnson PL, Bos K, Lari M, Bollongino R (2013). A revised timescale for human evolution based on ancient mitochondrial genomes. Curr Biol..

[24] Kimura M (1980). A simple method for estimating evolutionary rates of base substitutions through comparative studies of nucleotide sequences. J Mol Evol..

[25] Tamura K, Nei M (1993). Estimation of the number of nucleotide substitutions in the control region of mitochondrial DNA in humans and chimpanzees. Mol Biol Evol..

[26] Ingman M, Kaessmann H, Pääbo S, Gyllensten U (2000). Mitochondrial genome variation and the origin of modern humans. Nature..

[27] Andrews RM, Kubacka I, Chinnery PF, Lightowlers RN, Turnbull DM, Howell N (1999). Reanalysis and revision of the Cambridge reference sequence for human mitochondrial DNA. Nat Genet..

[28] Behar DM, Van Oven M, Rosset S, Metspalu M, Loogväli EL, Silva NM, et al. A “Copernican” reassessment of the human mitochondrial DNA tree from its root. Am J Hum Genet. 2012;90(4):675–84. 10.1016/j.ajhg.2012.03.002 PMC3322232 22482806

[29] Kolmogorov A (1933). Sulla determinazione empirica di una lgge di distribuzione. Inst Ital Attuari, Giorn..

[30] Smirnov N (1948). Table for estimating the goodness of fit of empirical distributions. Ann Math Stat..

[31] Meyer M, Fu Q, Aximu-Petri A, Glocke I, Nickel B, Arsuaga JL (2014). A mitochondrial genome sequence of a hominin from Sima de los Huesos. Nature..

[32] Noonan JP, Coop G, Kudaravalli S, Smith D, Krause J, Alessi J (2006). Sequencing and analysis of Neanderthal genomic DNA. Science..

[33] Endicott P, Ho SY, Stringer C (2010). Using genetic evidence to evaluate four palaeoanthropological hypotheses for the timing of Neanderthal and modern human origins. J Hum Evol..

[34] Rieux A, Eriksson A, Li M, Sobkowiak B, Weinert LA, Warmuth V (2014). Improved calibration of the human mitochondrial clock using ancient genomes. Mol Biol Evol..

[35] Gómez-Robles A. Dental evolutionary rates and its implications for the Neanderthal–modern human divergence. Sci Adv. 2019;5(5):eaaw1268. 10.1126/sciadv.aaw1268 PMC6520022 31106274

[36] Kumar S, Filipski A, Swarna V, Walker A, Hedges SB (2005). Placing confidence limits on the molecular age of the human-chimpanzee divergence. Proc Natl Acad Sci..

[37] Langergraber KE, Prüfer K, Rowney C, Boesch C, Crockford C, Fawcett K (2012). Generation times in wild chimpanzees and gorillas suggest earlier divergence times in great ape and human evolution. Proc Natl Acad Sci..

[38] Amster G, Sella G (2016). Life history effects on the molecular clock of autosomes and sex chromosomes. Proc Natl Acad Sci..

[39] Stone AC, Battistuzzi FU, Kubatko LS, Perry GH, Trudeau E, Lin H (2010). More reliable estimates of divergence times in Pan using complete mtDNA sequences and accounting for population structure. Phil Trans R Soc B Biol Sci..

