# Supplementary material for: Dating ancient splits in phylogenetic trees, with application to the human-Neanderthal split
Source: BMC Genom Data. 2024 Jan 2;25:4. doi: 10.1186/s12863-023-01185-8 (PMC10759710; doi:10.1186/s12863-023-01185-8)
Supplement: Supplementary file 1 — Additional file 1. [file 12863_2023_1185_MOESM1_ESM.pdf]

Supplementary Material: Dating Ancient Splits in Phylogenetic Trees, with Application to the Human-Neanderthal Split

Keren Levinstein Hallak[1] and Saharon Rosset[1,*]

[1]Department of Statistics and Operations Research, School of Mathematical Sciences, Tel-Aviv University, 6997801, Tel-Aviv, Israel
[*]Corresponding author, E-mail: saharon@tauex.tau.ac.il

1 Theoretical details

1.1 Proof of Lemma 1

Let $Y \sim \text{Pois}(\lambda)$ and Z be the parity of Y. Then $Z \sim Ber(\frac{1}{2}(1-e^{-2\lambda}))$.

Proof.

$$P(Z_i = 1) = \sum_{n=0}^{\infty} P(Y_i = 2n+1) = \sum_{n=0}^{\infty} e^{-\lambda} \frac{\lambda^{2n+1}}{(2n+1)!} =$$
$$= e^{-\lambda}\frac{1}{2}\left(\sum_{n=0}^{\infty}\frac{\lambda^n}{n!} - \sum_{n=0}^{\infty}\frac{(-\lambda)^n}{n!}\right) = \frac{e^{-\lambda}}{2}\left(e^{\lambda}-e^{-\lambda}\right) = \frac{1}{2}\left(1-e^{-2\lambda}\right).$$

□

1.2 Proof of Theorem 1

Denote the Fisher information matrix for the estimation problem above by $I \in \mathbb{R}^{(n+1,n+1)}$, where the first n indexes correspond to $\{\lambda_i\}_{i=1}^n$ and the last index $(n+1)$ corresponds to p. For clarity denote $I_{p,p} \doteq I_{n+1,n+1}, I_{i,p} \doteq I_{i,n+1}, I_{p,i} \doteq I_{n+1,i}$. Then:

$$I_{i,j} = 0, \quad I_{i,i} = \frac{1}{\lambda_i} + \frac{4p^2}{e^{4\lambda_i p}-1}, \quad I_{i,p} = I_{p,i} = \frac{4p\lambda_i}{e^{4\lambda_i p}-1}, \quad I_{p,p} = 4\sum_{i=1}^{n}\frac{\lambda_i^2}{e^{4\lambda_i p}-1}. \tag{9}$$

Consequently, an unbiased estimator $\hat{p}$ holds:

$$\mathbb{E}\left[(p-\hat{p})^2\right] \geq \left[4\sum_{i=1}^{n}\frac{\lambda_i^2}{e^{4\lambda_i p}-1+4p^2\lambda_i}\right]^{-1}. \tag{10}$$

If $\forall i = 1..n : \lambda_i = \lambda$, we can further simplify the expression:

$$\mathbb{E}\left[(p-\hat{p})^2\right] \geq \frac{e^{4\lambda p} - 1 + 4p^2\lambda}{4n\lambda^2}. \tag{11}$$

Proof. We calculate the second derivative of the log-likelihood. Denote:

$$\beta_i = -2\lambda_i p + j\pi Z_i, \quad \sigma(t) = \frac{e^t}{1+e^t},$$

then the first derivatives are given by:

$$\begin{aligned} \frac{\partial l}{\partial \lambda_i} &= -1 + \frac{X_i}{\lambda_i} + \frac{(-2p)\,(-1)^{Z_i}\exp\left(-2\lambda_i p\right)}{1 + (-1)^{Z_i}\exp\left(-2\lambda_i p\right)} \\ &= -1 + \frac{X_i}{\lambda_i} - 2p\sigma\left(-2\lambda_i p + j\pi Z_i\right) \\ &= -1 + \frac{X_i}{\lambda_i} - 2p\sigma\left(\beta_i\right), \end{aligned} \tag{12}$$

and

$$\frac{\partial l}{\partial p} = \sum_{i=1}^{n} \frac{(-2\lambda_i)\,(-1)^{Z_i}\exp\left(-2\lambda_i p\right)}{1 + (-1)^{Z_i}\exp\left(-2\lambda_i p\right)} = -2\sum_{i=1}^{n} \lambda_i \sigma\left(\beta_i\right). \tag{13}$$

The second derivatives are now given by:

$$\begin{aligned} \frac{\partial^2 l}{\partial \lambda_i \lambda_j} &= 0 \\ \frac{\partial^2 l}{\partial {\lambda_i}^2} &= -\frac{X_i}{\lambda_i^2} - 2p\left(-2p\right)\sigma\left(\beta_i\right)\left(1-\sigma\left(\beta_i\right)\right) = -\frac{X_i}{\lambda_i^2} + 4p^2\sigma\left(\beta_i\right)\left(1-\sigma\left(\beta_i\right)\right) \\ \frac{\partial^2 l}{\partial \lambda_i \partial p} &= -2p\left(-2\lambda_i\right)\sigma\left(\beta_i\right)\left(1-\sigma\left(\beta_i\right)\right) - 2\sigma\left(\beta_i\right) = 4p\lambda_i\sigma\left(\beta_i\right)\left(1-\sigma\left(\beta_i\right)\right) - 2\sigma\left(\beta_i\right) \\ \frac{\partial^2 l}{\partial p^2} &= \sum_{i=1}^{n} 4\lambda_i^2\sigma\left(\beta_i\right)\left(1-\sigma\left(\beta_i\right)\right) \end{aligned}$$

The expectation of these are given by:

$$\mathbb{E}\left[\sigma\left(\beta_i\right)\right] = \frac{1}{2}\left(1+\exp\left(-2\lambda_i p\right)\right)\frac{\exp\left(-2\lambda_i p\right)}{1+\exp\left(-2\lambda_i p\right)} + \frac{1}{2}\left(1-\exp\left(-2\lambda_i p\right)\right)\frac{(-1)\cdot\exp\left(-2\lambda_i p\right)}{1-\exp\left(-2\lambda_i p\right)} = 0$$

$$\begin{aligned}
\mathbb{E}\left[\sigma^2\left(\beta_i\right)\right] &= \frac{1}{2}\left(1+\exp\left(-2\lambda_i p\right)\right)\frac{\exp\left(-4\lambda_i p\right)}{\left(1+\exp\left(-2\lambda_i p\right)\right)^2} + \frac{1}{2}\left(1-\exp\left(-2\lambda_i p\right)\right)\frac{\exp\left(-4\lambda_i p\right)}{\left(1-\exp\left(-2\lambda_i p\right)\right)^2} = \\
&= \frac{1}{2}\exp\left(-4\lambda_i p\right)\left[\frac{1}{1+\exp\left(-2\lambda_i p\right)} + \frac{1}{1-\exp\left(-2\lambda_i p\right)}\right] = \frac{1}{e^{4\lambda_i p}-1}
\end{aligned}$$

$$\mathbb{E}\left[\frac{\partial^2 l}{\left(\partial\lambda_i\right)^2}\right] = E\left[-\frac{X_i}{\lambda_i^2} + 4p^2\sigma\left(\beta_i\right)\left(1-\sigma\left(\beta_i\right)\right)\right] = -\frac{1}{\lambda_i} - \frac{4p^2}{e^{4\lambda_i p}-1} = -I_{i,i}$$

$$\mathbb{E}\left[\frac{\partial^2 l}{\partial\lambda_i\partial p}\right] = E\left[4p\lambda_i\sigma\left(\beta_i\right)\left(1-\sigma\left(\beta_i\right)\right) - 2\sigma\left(\beta_i\right)\right] = -\frac{4p\lambda_i}{e^{4\lambda_i p}-1} = -I_{i,p}$$

$$\mathbb{E}\left[\frac{\partial^2 l}{\left(\partial p\right)^2}\right] = E\left[\sum_{i=1}^{n} 4\lambda_i^2\sigma\left(\beta_i\right)\left(1-\sigma\left(\beta_i\right)\right)\right] = -\sum_{i=1}^{n}\frac{4\lambda_i^2}{e^{4\lambda_i p}-1} = -I_{p,p}.$$

By CRB, for an unbiased estimator:

$$\begin{aligned}
\mathbb{E}\left[(p-\hat{p})^2\right] \geq [I^{-1}]_{p,p} &= \frac{1}{I_{p,p} - I_{p,i}I_{i,i}^{-1}I_{i,p}} \\
&= \left[\sum_{i=1}^{n}\frac{4\lambda_i^2}{e^{4\lambda_i p}-1} - \sum_{i=1}^{n}\frac{\frac{16p^2\lambda_i^2}{\left[e^{4\lambda_i p}-1\right]^2}}{\frac{1}{\lambda_i} + \frac{4p^2}{e^{4\lambda_i p}-1}}\right]^{-1} \\
&= \left[\sum_{i=1}^{n} 4\lambda_i^2 \frac{\left(e^{4\lambda_i p}-1\right)\left(\frac{1}{\lambda_i} + \frac{4p^2}{e^{4\lambda_i p}-1}\right) - 4p^2}{\left[e^{4\lambda_i p}-1\right]^2\left(\frac{1}{\lambda_i} + \frac{4p^2}{e^{4\lambda_i p}-1}\right)}\right]^{-1} \\
&= \left[4\sum_{i=1}^{n}\frac{\lambda_i^2}{e^{4\lambda_i p} - 1 + 4p^2\lambda_i}\right]^{-1}
\end{aligned}$$

□

1.3 Proof of Proposition 1

Proof. Following Equations 12, 13, we compare the first order derivatives to 0:

$$\frac{\partial l}{\partial \lambda_i} = -1 + \frac{X_i}{\lambda_i} - 2\hat{p}\frac{(-1)^{Z_i}e^{-2\lambda_i\hat{p}}}{\left(1+(-1)^{Z_i}e^{-2\lambda_i\hat{p}}\right)} = 0 \Rightarrow X_i = \hat{\lambda}_i + 2\hat{p}\hat{\lambda}_i\frac{(-1)^{Z_i}e^{-2\hat{\lambda}_i\hat{p}}}{\left(1+(-1)^{Z_i}e^{-2\hat{\lambda}_i\hat{p}}\right)}$$

$$\frac{\partial l}{\partial p} = -\sum_{i=1}^{n} 2\lambda_i\frac{(-1)^{Z_i}e^{-2\lambda_i\hat{p}}}{\left(1+(-1)^{Z_i}e^{-2\lambda_i\hat{p}}\right)} = -\sum_{i=1}^{n}\frac{\lambda_i}{\hat{p}}\left[-1+\frac{X_i}{\lambda_i}\right] = 0 \Rightarrow \sum_{i=1}^{n}\hat{\lambda}_i = \sum_{i=1}^{n} X_i.$$

Summing the first equation for every i and substituting the second equation results in the last part in Equation 6. □

1.4 Proof of Proposition 2

If $Y_i|X_i \sim Bin(X_i, p)$, then:

1. $Y_i \sim \text{Pois}(\lambda_i \cdot p)$, which justifies this approach.
2. $Z_i|X_i \sim Ber\left(\frac{1}{2}\left(1-(1-2p)^{X_i}\right)\right)$, so we can compute the likelihood of p without considering λ_i.
3. The maximum likelihood estimate of p given Z_i holds:

$$\sum_{i=1}^{n}\frac{X_i}{1+(-1)^{Z_i}(1-2p)^{-X_i}} = 0 \tag{14}$$

and the maximum likelihood estimate of p given $\sum_{i=1}^{n} Z_i$ holds:

$$\sum_{i=1}^{n}(1-2\hat{p})^{X_i} = n - 2\sum_{i=1}^{n} Z_i \tag{15}$$

Proof. Denote $q \equiv 1-p$. For item 1:

$$
\begin{aligned}
\Pr(Y_i = k) &= \sum_{n=k}^{\infty} \Pr(X_i = n) \cdot \Pr(Bin(n,p) = k) \\
&= \sum_{n=k}^{\infty} \frac{\lambda_i^n \cdot e^{-\lambda_i}}{n!} \cdot \Pr(\binom{n}{k})p^k q^{n-k} \\
&= \frac{(\lambda_i \cdot p)^k \cdot e^{-\lambda_i p}}{k!} \sum_{n=k}^{\infty} \frac{\lambda_i^{n-k} \cdot e^{-\lambda_i q}}{(n-k)!} \cdot q^{n-k} \\
&= \frac{(\lambda_i \cdot p)^k \cdot e^{-\lambda_i p}}{k!} \sum_{n=0}^{\infty} \frac{\lambda_i^{n} \cdot e^{-\lambda_i q}}{n!} \cdot q^{n} = \frac{(\lambda_i \cdot p)^k \cdot e^{-\lambda_i p}}{k!}.
\end{aligned}
$$

Now moving on to item 2:

$$
\begin{aligned}
\Pr(Z_i = 1|X_i) &= \Pr(Y_i \text{ is odd}|X_i), \quad Y_i|X_i \sim Bin(n = X_i, p) \\
(q+p)^n &= \Sigma_{k=0}^{n} \binom{n}{k} p^k q^{(n-k)} = P(Y_i \text{ is even}) + P(Y_i \text{ is odd}) \\
(q-p)^n &= \Sigma_{k=0}^{n} \binom{n}{k} (-p)^k q^{(n-k)} = P(Y_i \text{ is even}) - P(Y_i \text{ is odd})
\end{aligned}
$$

And summing up these two equations leads to:

$$
P(Y_i \text{ is even}) = \frac{1}{2}\left((q+p)^n + (q-p)^n\right) = \frac{1}{2}\left(1 + (1-2p)^n\right).
$$

Subsequently, the likelihood of Z_i is given by:

$$
\begin{aligned}
l(\vec{Z}; p) &= \prod_{i=1}^{n} \frac{1}{2}\left(1 + (-1)^{Z_i}(1-2p)^{X_i}\right) \\
L(\vec{Z}; p) &= \sum_{i=1}^{n} \log\left(1 + (-1)^{Z_i}(1-2p)^{X_i}\right) + Const
\end{aligned}
$$

33 Taking the derivative to 0:

$$
\frac{\partial L}{\partial p} = \sum_{i=1}^{n} \frac{-2(-1)^{Z_i} X_i (1-2p)^{X_i - 1}}{(1 + (-1)^{Z_i}(1-2p)^{X_i})} = \sum_{i=1}^{n} \frac{-2X_i}{((-1)^{Z_i}(1-2p)^{1-X_i} + 1 - 2p)} = 0, \tag{16}
$$

and division by $\frac{-2}{1-2p}$ yields the solution.

Now, according to Le Cam's theorem[1] [1], $\sum_{i=1}^{n} Z_i \sim \text{Pois}\left(\lambda = \sum_{i=1}^{n} \frac{1}{2}\left(1-(1-2p)^{X_i}\right)\right)$, and the likelihood is therefore:

$$L\left(\sum_{i=1}^{n} Z_i = m|\vec{X};p\right) = \lambda^m \frac{e^{-\lambda}}{m!}.$$

Now we look at the log-likelihood and take the derivative with respect to p to zero:

$$\begin{aligned}
l\left(\sum_{i=1}^{n} Z_i = m|\vec{X};p\right) &= m\log\lambda - \lambda + Const \\
&= m\log\left(\sum_{i=1}^{n}\frac{1}{2}\left(1-(1-2p)^{X_i}\right)\right) - \sum_{i=1}^{n}\frac{1}{2}\left(1-(1-2p)^{X_i}\right) + Const \\
\frac{\partial l}{\partial p} &= m\frac{\sum_{i=1}^{n} X_i(1-2p)^{X_i-1}}{\sum_{i=1}^{n}\frac{1}{2}\left(1-(1-2p)^{X_i}\right)} - \sum_{i=1}^{n} X_i(1-2p)^{X_i-1} \\
&= \left(\frac{m}{\sum_{i=1}^{n}\frac{1}{2}\left(1-(1-2p)^{X_i}\right)} - 1\right)\sum_{i=1}^{n} X_i(1-2p)^{X_i-1} = 0
\end{aligned}$$

Leading to the solution:

$$\sum_{i=1}^{n}(1-2\hat{p})^{X_i} = n - 2m = n - 2\sum_{i=1}^{n} Z_i$$

□

[1] More precisely:
$\sum_{k=0}^{\infty} |P(\sum_{i=1}^{n} Z_i = k) - \frac{1}{k!}(\sum_{i=1}^{n}\frac{1}{2}(1-(1-2p)^{X_i}))^k e^{-\sum_{i=1}^{n}\frac{1}{2}(1-(1-2p)^{X_i}}| < 2\sum_{i=1}^{n}\left(\frac{1}{2}(1-(1-2p)^{X_i})\right)^2$.

1.5 Proof of Proposition 3

Let $\lambda_i \sim \Gamma(\alpha,\beta)$, then the maximum a posteriori estimator of p holds:

$$\frac{\partial l}{\partial p} = \sum_{i=1}^{n} \frac{X_i+\alpha}{(-1)^{Z_i}\left(1+\frac{2p}{\beta+1}\right)^{X_i+\alpha}+1} = 0 \tag{17}$$

Subsequently, estimated values for α, β can be substituted for a numerical estimator for p.

Proof. We first compute the probability for each observation:

$$\begin{aligned}
\Pr\left(X_i = k, Y_i\, is\, even\right) &= \int_0^\infty P\left(\lambda_i=\lambda\right) P\left(X_i=k|\lambda_i=\lambda\right) P\left(Y_i\, is\, even|\lambda_i=\lambda\right) d\lambda \\
&= \int_0^\infty \lambda^{\alpha-1} e^{-\lambda\beta} \frac{\beta^\alpha}{\Gamma(\alpha)} e^{-\lambda} \frac{\lambda^k}{k!} \frac{1}{2}\left(1+e^{-2\lambda p}\right) d\lambda \\
&= \frac{\beta^\alpha}{2k!\Gamma(\alpha)} \left[\int_0^\infty \lambda^{\alpha-1+k} e^{-\lambda(\beta+1)} d\lambda + \int_0^\infty \lambda^{\alpha-1+k} e^{-\lambda(\beta+1+2p)} d\lambda\right] \\
&= \frac{\beta^\alpha}{2k!\Gamma(\alpha)} \Big[\frac{\Gamma(\alpha+k)}{(\beta+1)^{\alpha+k}} \underbrace{\int_0^\infty \lambda^{\alpha-1+k} e^{-\lambda(\beta+1)} \frac{(\beta+1)^{\alpha+k}}{\Gamma(\alpha+k)} d\lambda}_{=1} + \\
&\qquad \frac{\Gamma(\alpha+k)}{(\beta+1+2p)^{\alpha+k}} \underbrace{\int_0^\infty \lambda^{\alpha-1+k} e^{-\lambda(\beta+1+2p)} \frac{(\beta+1+2p)^{\alpha+k}}{\Gamma(\alpha+k)} d\lambda}_{=1} \Big] \\
&= \frac{\beta^\alpha \Gamma(\alpha+k)}{2k!\Gamma(\alpha)} \left[\frac{1}{(\beta+1)^{\alpha+k}} + \frac{1}{(\beta+1+2p)^{\alpha+k}}\right] \\
&= \frac{\Gamma(\alpha+k)}{2k!\Gamma(\alpha)} \left[\left(\frac{\beta}{\beta+1}\right)^\alpha \left(\frac{1}{\beta+1}\right)^k + \left(\frac{\beta}{\beta+1+2p}\right)^\alpha \left(\frac{1}{\beta+1+2p}\right)^k\right]
\end{aligned}$$

Hence, the likelihood is given by:

$$\begin{aligned}
&L\left(\vec{X}, \vec{Z}; p, \alpha, \beta\right) = \\
&= \prod_{i=1}^{n} \frac{\Gamma(\alpha+k)}{2k!\Gamma(\alpha)} \left[\left(\frac{\beta}{\beta+1}\right)^\alpha \left(\frac{1}{\beta+1}\right)^{X_i} + (-1)^{Z_i} \left(\frac{\beta}{\beta+1+2p}\right)^\alpha \left(\frac{1}{\beta+1+2p}\right)^{X_i}\right]
\end{aligned}$$

and the log-likelihood:

$$l\left(\vec{X},\vec{Z};p,\alpha,\beta\right)=\sum_{i=1}^{n}\log\frac{\Gamma\left(\alpha+X_i\right)}{X_i!\Gamma\left(\alpha\right)}+\alpha log\beta-(X_i+\alpha)\log(\beta+1)+\log\left[1+(-1)^{Z_i}\left(\frac{\beta+1}{\beta+1+2p}\right)^{X_i+\alpha}\right]$$

Now comparing the derivative with respect to p to zero:

$$\frac{\partial l}{\partial p}=\sum_{i=1}^{n}\frac{-\frac{2}{\beta+1}\left(-1\right)^{Z_i}\left(X_i+\alpha\right)\left(1+\frac{2p}{\beta+1}\right)^{-X_i-\alpha-1}}{1+\left(-1\right)^{Z_i}\left(1+\frac{2p}{\beta+1}\right)^{-X_i-\alpha}}=0$$

□

2 Simulation details and additional experiments

2.1 K2P and TN93 simulations

We extracted the parameters of the rate matrix from Phylotree's data and simulated a tree in the same total branch length as Phylotree, with branches short enough to contain an average of less than one base change along the sequence (the branches' length was set as $t = 1.5e - 05$ when 1 is the total length of Phylotree's branches). The rate matrix at each site was scaled by the observed substitution rate λ_i which is the total number of substitutions per site observed along Phylotree. For sites with $\lambda_i = 0$ we used instead $\lambda_i = \epsilon$ with ϵ chosen as explained in the following Supplementary subsection 2.2. Then, using the same rate matrix, we simulated sequences with a predefined distance p from the RSRS and assessed p using our methods.

2.2 Phylogenetic tree simulations

The rate parameter for sites with no transitions along the tree is denoted as ϵ, and we estimate it using the following simulation-based method. To generate $\vec{\lambda}$, we use the following

equation:

$$\min D = \sup_x |F(\vec{X}_{\text{mtDNA}}) - F(\vec{X})| \quad s.t. \quad \lambda_i = \begin{cases} X_{\text{mtDNA},i} & X_{\text{mtDNA},i} \neq 0 \\ \epsilon & X_{\text{mtDNA},i} = 0 \end{cases} \tag{18}$$

The value of ϵ is chosen to minimize the Kolmogorov–Smirnov statistic. Figure S1 shows a simulation of $D(\epsilon)$, with the mean of 1,000 runs for each ϵ value. The minimum value of D is obtained for $\epsilon = 0.0913$ (marked in red).

To make the simulated data closer to the real data, we also model transversions. We estimate the transversion rate per site in the same manner as the transition rate, using the Kolmogorov–Smirnov statistic to account for sites with no transversions. This results in $\epsilon_{\text{transversion}} = 0.0149$. To determine the nucleotide at a given site, we sample whether an odd number of transversions have occurred. If so, a random nucleotide is sampled from the two available transversion options. The resulting sequence is then input into BEAST2, but our methods still use only the sites without observed transversions. Finally, the analysis is limited to the gene regions in the genome (11,341 sites).

2.3 BEAST2 run parameters

The sequences used in this work were aligned using mafft [2], and the 11.3 kb of protein-coding genes were extracted and used for the analysis. The analysis followed the approach described in [3], where the best fitting clock and tree model for the tree were identified using path sampling with the model selection package in BEAST2 [4, 5, 6]. Each model test was run with 40 path steps, a chain length of 25 million iterations, an alpha parameter of 0.3, a pre-burn-in of 75,000 iterations, and an 80% burn-in of the entire chain. The mutation rate was set to 1.57 x 10E-8 and a normal distribution (mean: mutation rate, sigma: 1.E-10) was used for a strict clock model [7]. The TN93 substitution model [8] was

Figure S1: Kolmogorov–Smirnov statistic as a function of ϵ.

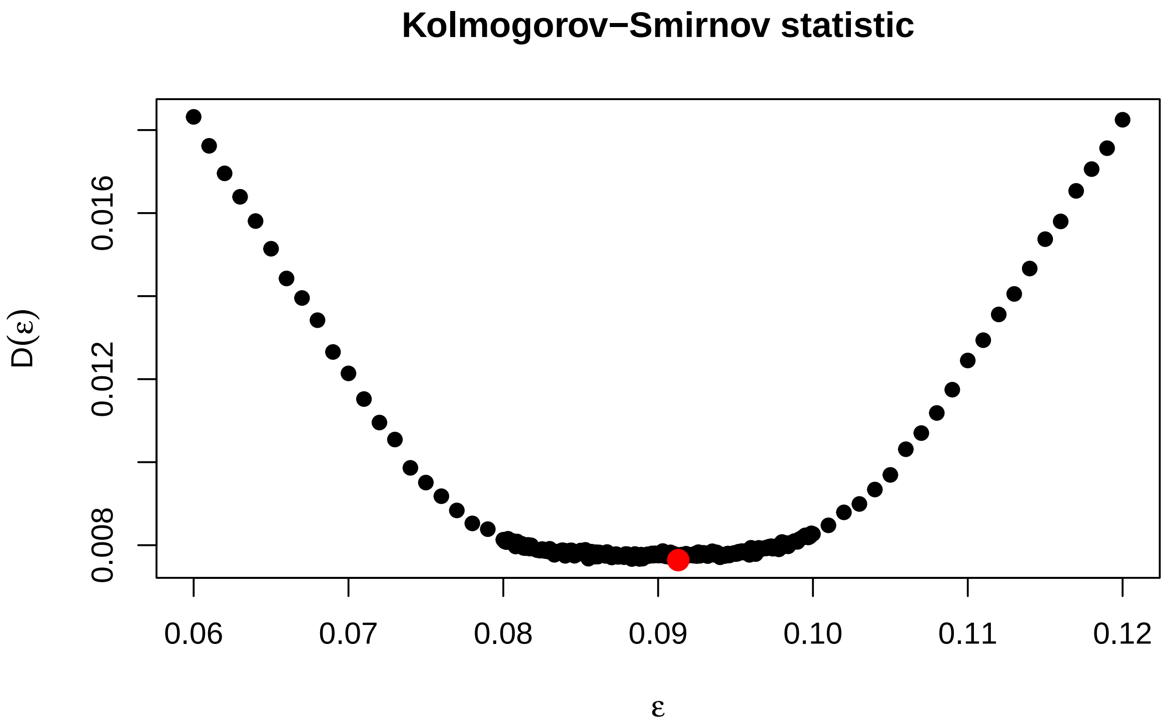

Figure S1. We performed 1,000 runs for each value of epsilon. The minimal $D(\epsilon)$ is marked red and equals $\epsilon = 0.0913$.

used for all models. The tree was calibrated with carbon dating data from ancient humans and Neanderthals, where available [9, 7, 10], and modern samples were set to a date of 0. All simulations were run with 4 gamma rate categories, 10,000,000 iterations, and a pre-burn-in of 1,000,000 iterations.

Figure S2: Comparison of estimators applied on a simulated long branch without a fixed tree topology for BEAST2.

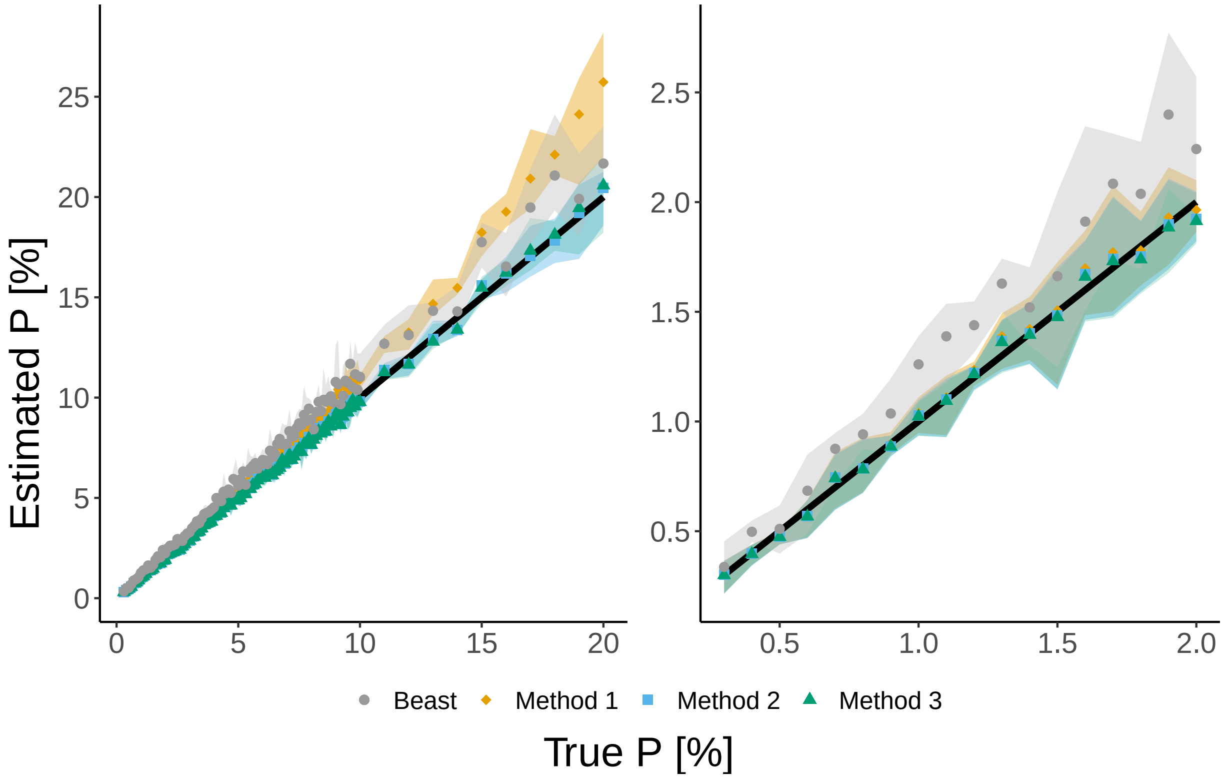

Figure S2. Comparison of our methods with BEAST2 estimator using simulated data. The right plot shows a zoom-in view of the left plot, focusing on values of p between 0 and 20%. Each point in the plot represents the average of 5 runs, while the shaded regions indicate the range of estimations obtained. BEAST2 is not using a fixed tree topology here.

Figure S3: Estimation errors for various BEAST2 estimators compared to Method 2.

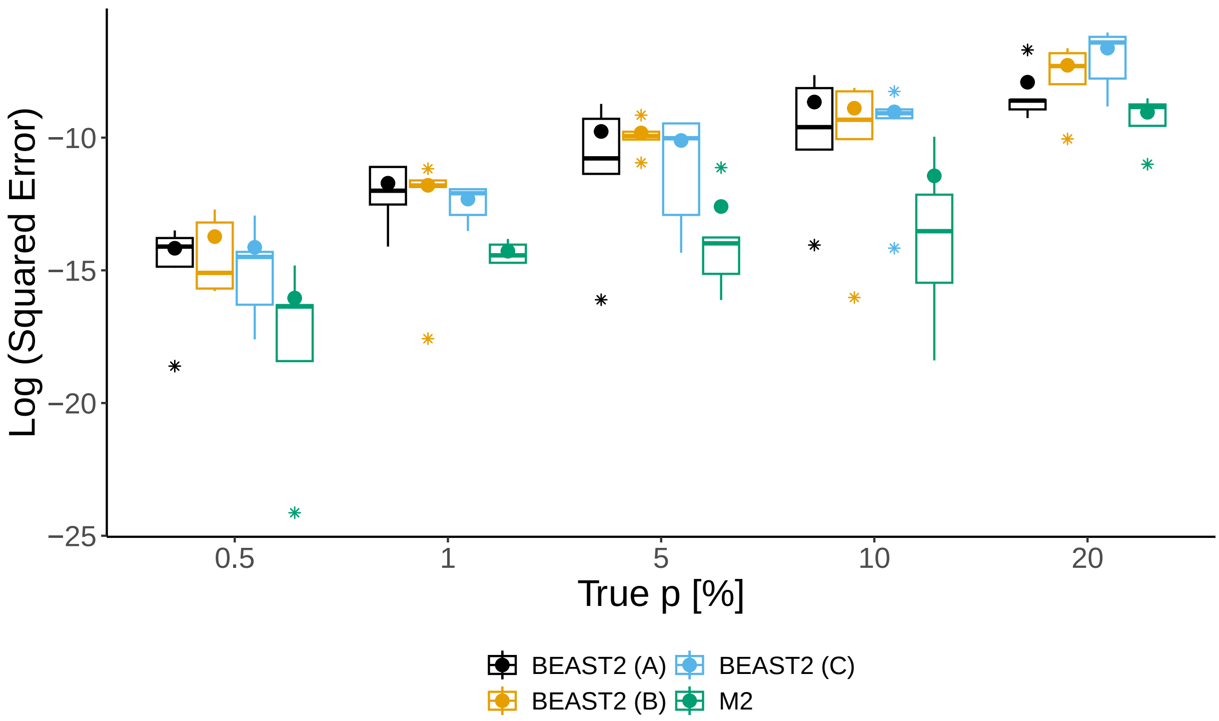

Figure S3. Box-plot of the log squared estimation errors of the different BEAST2 simulations for selected values of p, expressed as a percentage of the total length of Phylotree's edges (outliers are marked with $*$). The simulations were run 5 times for each value of p. BEAST2 (A): BEAST2 simulation with no fixed tree topology. BEAST2 (B): BEAST2 simulation with a fixed tree topology. BEAST2 (C): BEAST2 simulation with a fixed tree topology and 50 additional human sequences. M2: Method 2 which obtained the lowest squared error in our simulations. We performed a one-sided paired Wilcoxon signed rank test on every pair of simulation variations, correcting for multiple comparisons using the Bonferroni correction. Our results show no significant improvement in the squared error between the various BEAST2 simulations. However, when comparing to Method 2 the test shows that Method 2 has the lowest squared error.

Figure S4: Comparison of estimators applied on a simulated long branch with two ti/tv ratios

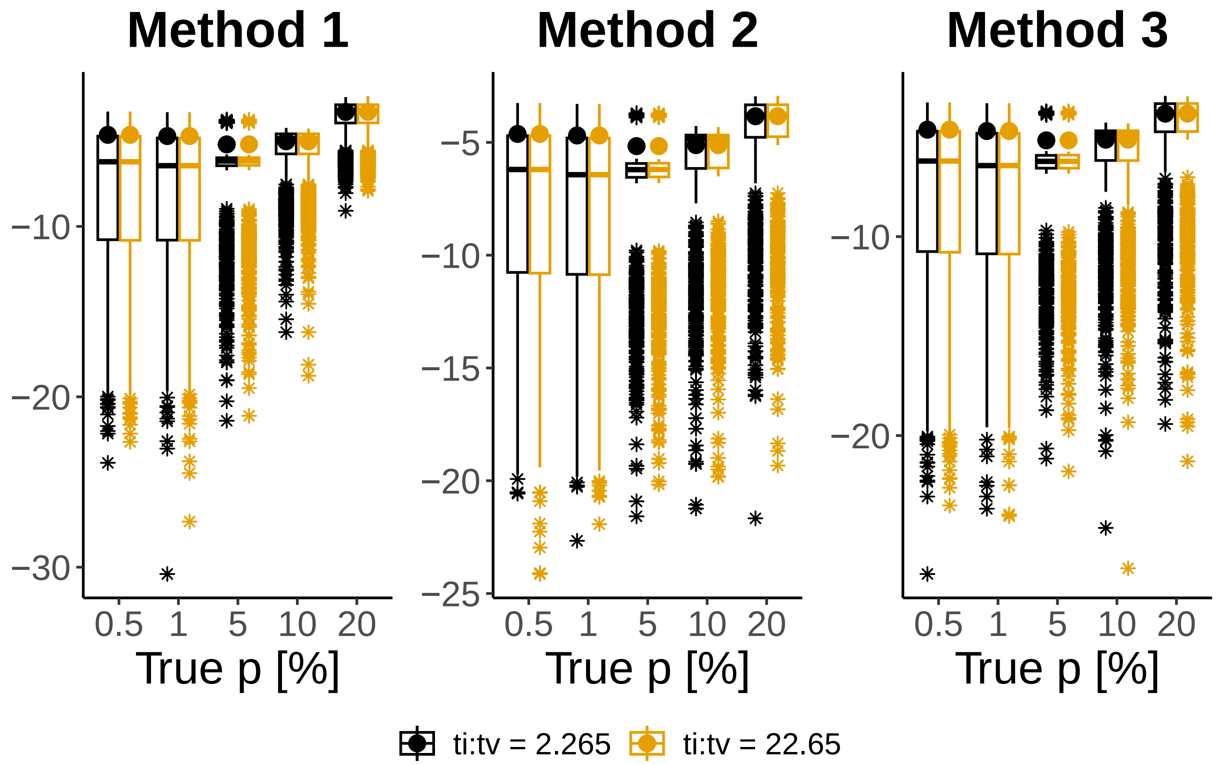

Figure S4. Box-plot of the log squared estimation errors of our methods for different fixed ti/tv ratios for selected values of p, expressed as a percentage of the total length of Phylotree's edges (outliers are marked with $*$). The simulations were run $1,000$ times for each value of p. For all methods, a lower ti/tv ratio does not change the expectation of the estimator.

References

[1] Le Cam L. An approximation theorem for the Poisson binomial distribution. Pacific Journal of Mathematics. 1960;10(4):1181-97.

[2] Katoh K, Toh H. Parallelization of the MAFFT multiple sequence alignment program. Bioinformatics. 2010;26(15):1899-900.

[3] Vernot B, Zavala EI, Gómez-Olivencia A, Jacobs Z, Slon V, Mafessoni F, et al. Unearthing Neanderthal population history using nuclear and mitochondrial DNA from cave sediments. Science. 2021;372(6542):eabf1667.

[4] Kass RE, Raftery AE. Bayes factor and model uncertainty. Journal of the American Statistical Association. 1995;90(430):773-95.

[5] Baele G, Li WLS, Drummond AJ, Suchard MA, Lemey P. Accurate model selection of relaxed molecular clocks in Bayesian phylogenetics. Molecular biology and evolution. 2012;30(2):239-43.

[6] Leaché AD, Fujita MK, Minin VN, Bouckaert RR. Species delimitation using genome-wide SNP data. Systematic biology. 2014;63(4):534-42.

[7] Fu Q, Mittnik A, Johnson PL, Bos K, Lari M, Bollongino R, et al. A revised timescale for human evolution based on ancient mitochondrial genomes. Current biology. 2013;23(7):553-9.

[8] Tamura K, Nei M. Estimation of the number of nucleotide substitutions in the control region of mitochondrial DNA in humans and chimpanzees. Molecular biology and evolution. 1993;10(3):512-26.

[9] Posth C, Wißing C, Kitagawa K, Pagani L, van Holstein L, Racimo F, et al. Deeply divergent archaic mitochondrial genome provides lower time boundary for African gene flow into Neanderthals. Nature communications. 2017;8(1):1-9.

[10] Wood RE, Higham TF, De Torres T, Tisnérat-Laborde N, Valladas H, Ortiz JE, et al. A new date for the Neanderthals from El Sidrón Cave (Asturias, northern Spain). Archaeometry. 2013;55(1):148-58.
